# Engagement With Tailored Physical Activity Content: Secondary Findings From the Families Improving Together for Weight Loss Randomized Controlled Trial

**DOI:** 10.2196/42581

**Published:** 2023-04-12

**Authors:** Allison M Sweeney, Dawn K Wilson, Kenneth Resnicow, M Lee Van Horn, Heather Kitzman

**Affiliations:** 1 Department of Biobehavioral Health & Nursing Science College of Nursing University of South Carolina Columbia, SC United States; 2 Department of Psychology University of South Carolina Columbia, SC United States; 3 Department of Health Behavior & Health Education University of Michigan Ann Arbor, MI United States; 4 Department of Individual, Family, and Community Education University of New Mexico Albuquerque, NM United States; 5 Baylor Scott and White Health Baylor Scott & White Health and Wellness Center Dallas, TX United States

**Keywords:** tailoring, eHealth, African Americans, physical activity, weight loss

## Abstract

**Background:**

Web-based tailored interventions offer rich opportunities for improved access to and personalization of behavioral interventions. However, despite the promise of this approach, the engagement and underrepresentation of minority groups remain major issues.

**Objective:**

This study evaluated whether engagement (log-in status and log-in duration) with different types of tailored behavioral content from the Families Improving Together for weight loss web-based intervention was associated with changes in moderate to vigorous physical activity (MVPA) among African American families with overweight or obesity.

**Methods:**

Parent-adolescent dyads were randomized to a web-based tailored intervention or web-based health education comparison program. The web-based intervention (N=119) was completed by parents and targeted 6 weight-related behaviors to support their adolescent children’s weight loss goals (session contents included energy balance, fast food, fruits and vegetables, physical activity [PA], sedentary behavior, and sweetened beverages). MVPA was measured using accelerometers at baseline and after the intervention.

**Results:**

Using a hierarchical approach, the log-in status and duration for each web-based session were used to evaluate the additive effects of engagement with different types of tailored behavioral content on MVPA after the web-based intervention. Among parents, logging in to the PA session was not associated with greater MVPA (*B*=−12.561, 95% CI −18.759 to −6.367), but MVPA increased with greater log-in duration for the PA (*B*=0.008, 95% CI 0.004-0.012) and sedentary behavior (*B*= 0.008, 95% CI 0.004-0.012) sessions. These results suggest that parents who logged in to the PA session had lower MVPA, but MVPA increased with greater log-in duration for the PA and sedentary behavior sessions. These associations remained even after accounting for engagement with other content sessions. However, these engagement effects did not translate to the adolescents.

**Conclusions:**

The results of this study highlight the need to disentangle the impact of engagement with different tailored content to improve the efficacy of tailored web-based interventions, especially for promoting PA in African American families.

**Trial Registration:**

ClinicalTrials.gov NCT01796067; https://clinicaltrials.gov/ct2/show/NCT01796067

## Introduction

### Background

Childhood obesity remains a major public health concern, especially for racial and ethnic minority youth who experience significantly higher rates of overweight and obesity than their non-Hispanic White peers [1,2]. Approximately 80% of adolescents with obesity continue to have obesity as adults [3], leading to an increased risk for a range of health problems, including breathing problems, cardiovascular disease, type 2 diabetes, cancer, and premature death [4-6]. The burden of chronic disease disproportionately affects African American families, as evidenced by large and consistent disparities in the leading causes of death (eg, cancer and cardiovascular disease) across the adult life span [7,8]. These health inequities are driven by a variety of social, environmental, and structural factors, including structural racism, chronic stressors, and a lack of neighborhood resources [9-11]. Such findings highlight the importance of intervening during adolescence to promote positive changes in weight-related behaviors (eg, physical activity [PA]) while also addressing parental stress and family support [12,13].

Efforts to understand the best practices for engaging African American families in behavioral weight loss programs have focused on reducing barriers to participation, such as distrust, the lack of transportation, and the lack of interest [14]. One approach for overcoming these barriers is through tailored web-based interventions, which compared with traditional in-person programs, offer opportunities for increased access, personalization, and in-the-moment feedback [15]. Tailored web-based interventions involve measuring a range of individual factors, such as traits, beliefs, and past behaviors, to create highly personalized intervention materials. Compared with targeted approaches (ie, adapting an intervention to a particular group), individually tailored interventions, especially those that integrate sociocultural values and norms, are theorized to be a more effective approach because they enhance the personal relevancy of health behavior change [16] and promote greater mental elaboration [17]. However, despite the promise of tailored web-based interventions to improve access and engagement, the lack of representation of racial and ethnic minority groups remains a major issue for web-based interventions [15,18,19].

### Objective

This study aimed to address this gap by evaluating a multitheoretical approach to engaging African American families in a tailored web-based weight loss program, including a novel focus on positive parenting practices. The Family Systems Theory highlights the importance of nurturance and positive parenting practices for promoting positive family interactions [20]. Complementing this approach, the Self-Determination Theory [21] proposes that offering adolescents choice and input around health behaviors is essential for promoting enjoyment and autonomous motivation, further highlighting the importance of parenting practices that build autonomy support and intrinsic motivation. Although some web-based interventions have targeted parents as agents of change, previous studies have had mixed success [22,23]. This may be because previous web-based weight loss interventions have drawn from other theories (eg, the Transtheoretical Model) and have not included a primary focus on parenting [22], which may be important for engaging African American families in health behavior change.

Another approach to improving engagement among African American families is through deep-level cultural tailoring. Previous research has demonstrated that interventions that are culturally adapted lead to improved health outcomes among racial minority groups [24,25]. Importantly, among web-based studies, most previous interventions for African American families have focused on surface-level approaches by including culturally relevant images and content (eg, information about foods commonly eaten by African American families) [26,27]. Although surface-level approaches enhance the appearance of cultural relevancy, deep-level cultural tailoring that addresses social, cultural, and psychological characteristics (eg, values, cultural beliefs, and norms) can promote more autonomous sources of motivation [28], which is an important predictor of long-term health behavior change [29]. There are considerable differences in the racial and ethnic identity among African American families (eg, racial salience, multicultural beliefs, and Afrocentric values) [30]. Considering this variability, a deep-level culturally tailored approach allows for enhanced personalization by measuring family members’ personal and cultural views to design intervention materials that are tailored to match their specific views.

This study expands upon past web-based tailored interventions by integrating several novel tailoring components, including positive parenting (parenting style, monitoring, and communication), deep-level cultural tailoring variables (ethnic identity, spirituality, cultural, and personal values), and other constructs (autonomy support, motivation, and social support). Specifically, drawing from this novel multitheoretical framework, this study seeks to better understand the effects of engagement with different types of tailored behavioral content (eg, PA vs diet-related content). Increasingly, researchers find that engagement (eg, total log-in duration and dose) is related to the efficacy of web-based interventions [31,32]. However, few studies have compared engagement with different types of tailored behavioral content, especially among African American families who remain underrepresented in web-based interventions [15,18,19].

This study presents the secondary findings from the Families Improving Together (FIT) for weight loss trial, which compared a parent-focused 8-week web-based tailored intervention and a web-based health education program among African American families with adolescent children with overweight or obesity [33,34]. Follow-up analyses from the FIT trial indicated that parents who completed >70% of the web-based tailored intervention sessions engaged in greater moderate to vigorous physical activity (MVPA), with a similar trend in adolescents [34]. In light of these findings, this study aimed to better understand the effects of engagement with different types of tailored behavioral content in web-based interventions.

Thus, the primary aim of this study was to evaluate whether engagement with different types of tailored behavioral sessions from our multitheoretical web-based tailored intervention (eg, PA vs diet) was associated with changes in MVPA among African American parents and their adolescent children. We hypothesized that there would be a behavior-specific pathway such that engagement with tailored PA-related content would be associated with greater MVPA after the web-based intervention.

## Methods

### Overview of the FIT Trial

The FIT trial was a randomized group cohort study that tested the efficacy of a motivational plus family-based weight loss (M+FWL) intervention compared with a comprehensive health education control group for reducing BMI among African American adolescents with overweight or obesity and their caregivers [33,34]. The trial was registered on ClinicalTrials.gov (NCT01796067). The FIT intervention used a multitheoretical approach, including integrating components from the Family Systems Theory (eg, positive parenting and communication skills), [20], Self-Determination Theory (eg, autonomy support and motivation) [21], Social Cognitive Theory (eg, goal-setting and self-monitoring) [35], and cultural adaptations for African American families. Participants were recruited from Columbia, South Carolina, and surrounding areas (within 60 miles) between 2013 and 2018, with the final cohort completing data collection in Spring 2019.

In phase 1, families were randomized to an 8-week face-to-face group-based M+FWL or comprehensive health education program [33,34,36,37]. In phase 2, families were rerandomized to an 8-week web-based tailored intervention or web-based comparison program, resulting in a 2 (group treatment) × 2 (web-based treatment) factorial design. A full description of the intervention and primary results has been published previously [34]. This study focuses on the web-based tailored intervention condition only to evaluate whether engagement with different types of tailored content related to changes in PA. The web-based comparison program included links to websites with general health information on other unrelated topics (eg, tobacco prevention, oral hygiene, and sleep) and did not include any tailored components. Because the web-based comparison program included links to external websites, we were able to record whether a link was clicked but not the amount of time spent on the external websites. As a result, we were not able to make direct comparisons in terms of log-in duration between the web-based comparison program and web-based intervention. Therefore, for the purposes of this study, we focused specifically on the web-based tailored intervention to investigate whether engagement with the different tailored sessions related to differences in PA.

### Participants

Participants were recruited through community partnerships, culturally relevant advertisements, and community events [38]. A total of 241 families (parent-teen dyads) were randomized to a web-based program, with 119 (49.4%) in the web-based intervention. The eligibility criteria included having an African American adolescent (aged 11-16 years) with overweight or obesity (BMI ≥85th percentile for age and sex), having a caregiver willing to participate, and having internet access at home. This information was self-reported by the caregiver during an initial phone-based screener (including the race of the caregiver and race of the adolescent). At the enrollment, adolescent BMI was measured using a height board and a medical-grade scale to confirm eligibility. Adolescents with overweight were included because they were considered to be at a high risk for obesity. Adolescents were excluded if they had a medical or psychiatric condition that would interfere with PA or dietary behaviors, were currently participating in a weight loss program, or were taking medication that could interfere with weight loss.

### Ethics Approval

This study was reviewed and approved by the Institutional Review Board of the University of South Carolina (approval no. Pro00016136). The parents signed informed consent forms, and adolescents provided verbal assent.

### Overview of the Web-Based Program

After completing the phase 1 of the intervention, all participants (adolescents and parents) completed a tailoring questionnaire before being randomized to the web-based tailored intervention or the web-based comparison program. The tailoring questionnaire was used in the intervention to provide an individually tailored program that integrated the perspectives of both parents and their adolescent children, whereas those in the web-based comprehensive program received generalized health information. Parents were targeted to participate in the web-based program, as the intervention was designed to promote positive parenting skills to support adolescent weight loss. Both web-based programs consisted of 8 weekly web-based sessions delivered through a secure website. At the start of each week, parents received an automated text reminder to complete the week’s sessions. A research assistant (blinded to condition) provided additional reminders later in the week (by phone, email, or text) and was available to provide technical support as needed. The web-based program was developed in collaboration with the University of Michigan Center for Health Communication Research.

### Web-Based Tailored Intervention

#### Overview of the Program

**Figure 1:**
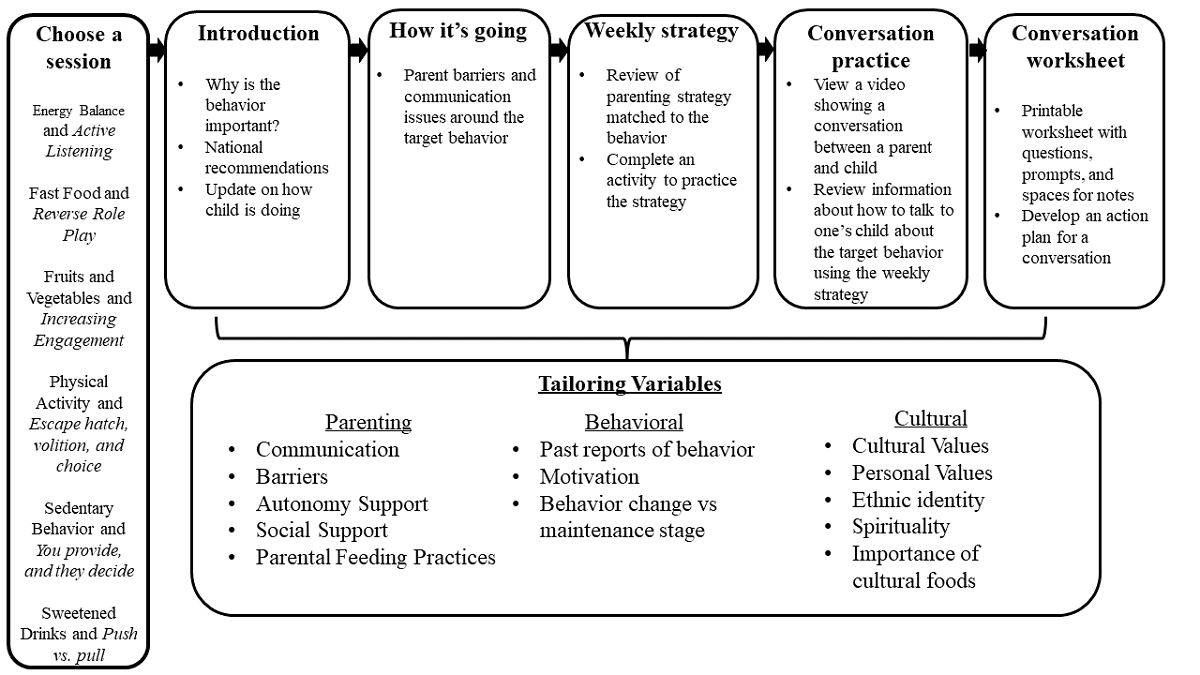
Overview of the content and tailoring strategies used in the web-based intervention.

The web-based intervention was tailored on numerous constructs from both parents and adolescents, including parenting, behavior, and cultural variables (Figure 1). Drawing from the Self-Determination Theory, both the tone and content of the program were designed to promote autonomous motivation by highlighting volition and autonomy (ie, emphasizing the choice and personal meaning of health behavior change for both parents and adolescents). For example, tailored messages used autonomy-supportive language, such as “you might consider” or “how might this benefit you?” rather than controlling language (eg, “you must” or “you have to”). To promote autonomy-supportive parenting practices, each session introduced a new parenting skill that was paired with a weight-related behavior: for example, (1) energy balance and meeting a calorie goal/*active listening*; (2) fast food/*reverse role* *play*; (3) fruits and vegetables/*increasing engagement*; (4) PA/*escape hatch, volition, and choice*; (5) screen time/*you provide and they decide*; and (6) sweetened drinks/*push versus pull*.

The program began with a welcome session in week 1, which included an overview of the program and an introduction of the 6 content sessions. In the first week, parents developed a calorie-related goal for their adolescent children and completed a tailored autonomy-supportive parenting exercise to assess their current parenting styles and practices. In the following weeks, each session followed the same basic structure completing a check-in on goals, providing feedback on the adolescents’ progress, completing a content session, and developing an action plan for the upcoming week. Each session began with a check-in survey, in which parents reported their adolescent children’s behavior in the 6 targeted areas and their progress while meeting their calorie goal. This information was used to provide parents with ongoing tailored feedback about their adolescents’ progress (compared with previous reports). Parents were then guided to select one of the 6 content sessions to complete. The order of these sessions was tailored based on the adolescents’ progress and their willingness to change each behavior such that behaviors that were more challenging and those that the adolescents were willing to work on appeared first, followed by behaviors that were less challenging or those that the adolescents were less willing to change. However, parents were free to select the session they wanted to complete in a given week. In the final session during week 8, the content focused on ways in which parents could continue to support their adolescent children with their weight loss goals, including reviewing the autonomy-supportive parenting strategies and tips for applying them to the 6 targeted health behaviors. Parents completed one last check-in and received feedback on their adolescents’ progress across the 8-week program.

#### Description of Content Sessions

Each content session involved behavior-specific content, a check-in on barriers to autonomy-supportive parenting, introduction of a new positive parenting practice, a worksheet to practice parenting skills, conversation practice with a brief video and worksheet, and the development of an action plan for the upcoming week. The behavior-specific content included information about why the behavior was important for weight loss and current guidelines, as well as tailored feedback about their adolescent children’s progress with that behavior since starting the web-based program. Information and feedback were tailored around the parents’ personal and cultural values and the adolescents’ previously reported motivation for behavior change.

Next, parents reported their current barriers to implementing autonomy-supportive parenting (eg, “What gets in the way of you being autonomy supportive?”) and received tailored feedback, which was based on parent-reported barriers to communication, parent- and adolescent-reported communication, the parents’ cultural views, and the adolescents’ report of perceived social support around health behaviors from their parents. Parents were then introduced to the new positive parenting strategy, including a definition and when or how to use it. Parents completed a worksheet to practice implementing the strategy (eg, reframing the controlling language with an autonomy-supportive language). They then watched a brief video with a parent-adolescent dyad (tailored to match the race and sex of the participant and their adolescent) that provided a real example of how to implement the parenting strategy in a conversation about the targeted behavior (Table 1 provides an example).

**Table 1:** Example script from brief video demonstrating conversation practice (positive parenting practice paired with discussion of a weight-related behavior).

Speaker	Dialogue
Narrator	“This video shows two examples of a mother and daughter talking about sweet drinks. In the first example, Mom is pushing, or telling, Brianna to cut back on sweet drinks. In the second example, Mom uses a pulling, or asking, approach.”
Mother	(Mom is preparing dinner while Brianna does her homework at the kitchen table. Mom addresses Briana with a bossy tone of voice) “Brianna, I’d like to talk to you about something. Do you have a minute?”
Daughter	(Not happy to be interrupted) “Um, I’m kind of in the middle of my homework. What do you want?”
Mother	“I’ve been thinking about how we can get healthier. I’ve decided that it’s really important for us to stop drinking so much soda and juice. It’s not good for our health—we should be drinking water instead. I’m going to stop buying sweet drinks so we won't have them in the house anymore. I don’t want you to have any when you're with your friends either, okay? Trust me, I know what’s best.”
Daughter	(Exasperated—can’t believe this is what Mom interrupted her to talk about) “Seriously? That’s what you wanted to talk about? I really don’t see what the big deal is. All my friends drink soda. Besides, it’s so boring drinking water all the time! I’m not a baby, and I’m not just going to stop drinking soda and juice.”
Narrator	“Using a pushy OR CONTROLLING tone doesn’t seem to work with Brianna. In the next example, let’s see what might have happened if Mom tried a pulling approach instead. Notice how this time Brianna is encouraged to think about her decisions rather than just being told what to do.”
Mother	(Walks over to the table and sits down with Brianna) “Brianna, I’d like to talk to you about soda and juice. As a family, we’re trying to be healthier and I’m wondering what thoughts you have about soda and juice.”
Daughter	“I don’t know. I don’t really see what the problem is. All my friends drink soda.”
Mother	“Let’s talk about this. What ideas do you have about why sweet drinks may not be such a healthy choice?”
Daughter	“Well, they do have a lot of calories, right?”
Mother	“That’s true. They have lots of sugar and calories. How might that be affecting you?”
Daughter	“Well, I guess it could keep me from losing weight.”
Mother	“Yeah, that could be. All those empty calories make it easy to gain weight. So how might we go about drinking less of these sugary drinks?”
Daughter	“I don’t really know. I guess I could try to cut back.”
Mother	“That’s a great idea. How much do you think is reasonable each week?”
Daughter	“Um, maybe I could limit myself to soda every other day?”
Mom	“That would be a really great start! I know you said drinking water is boring. But if you add a slice of lemon or lime to it, it has more flavor. Would you be willing to try that?”
Daughter	“Yeah, I could give that a try.”
Mother	“What other ways can you think of to cut back on sugary drinks?”
Daughter	“I sometimes see my teacher drinking flavored seltzer. Could I drink that?”
Mother	“Sure! That’s a great idea. I'll buy a few flavors and you can see which ones you like.”
Narrator	“Brianna responds better when her mom uses pull language. Instead of telling Brianna what she must do, Mom asks questions and gets Brianna’s opinions and ideas about sweet drinks. Brianna is more willing to consider cutting back when she is allowed to be involved in the decisions.”

After watching the video, parents completed a conversation worksheet and set an action plan for the upcoming week, which was tailored based on whether it was a behavior that required change or maintenance, parent- and adolescent-reported communication, parents’ views on gender roles and cultural values, and adolescents’ personal values. The goal of the conversation worksheet was to prepare parents to talk to their adolescent children about the target behavior (eg, “Why would your child want to work on this behavior?” “What is going well with this behavior?” and “What has been difficult?”) and implement the positive parenting strategy (eg, “How will you show them you are listening?” and “How will you monitor your child’s progress in an autonomy-supportive way?”). The worksheet included a printable page with discussion prompts and space for notes to guide the conversations with their adolescent children.

### Measures

All measures were collected by trained measurement staff (blinded to condition) at baseline, postintervention time point (8 weeks), and post–web-based intervention time point (16 weeks).

#### Log-in Status and Duration

For the web-based tailored intervention only, program use data were used to evaluate whether participants logged into a given session and how long they spent logged in. Log-in status was defined based on whether a participant logged into a given session at all. The log-in duration was calculated based on the time stamp for logging in, requesting a page, and logging out. If a participant did not interact with the website for >30 minutes, then the session ended at the time the participant requested the last page. In a given week, the participants were able to log in and out of a content session multiple times to complete the session. Thus, log-in duration reflects the total accumulated time spent on a content session in a given week.

#### BMI Measurements

Height (cm) and weight (kg) measurements were obtained using a Seca 880 digital scale and Shorr height board. BMI values were calculated using the standard BMI formula (kg/m^2^). Adolescent BMI scores were converted to BMI *z* scores using the NutStat (EpiInfo) program based on Centers for Disease Control and Prevention sex-specific 2000 reference curves.

#### PA Measurements

MVPA measurements were obtained using 7-day estimates from omnidirectional Actical accelerometers. For the adolescents, cut points developed for use in youth populations were used [39] with 60-second epochs to classify accelerometer counts as MVPA (counts >1500). A total of 20 consecutive 0 counts were coded as nonwear time for adolescents [40]. For parents, previously validated cutoff points [41] with 60-second epochs were used to classify accelerometer counts as MVPA (counts >1535), and 60 consecutive 0 counts were coded as nonwear time [42].

#### Analysis Plan

To evaluate the effect of engagement with different types of tailored behavioral content, a hierarchical mixed model approach was used. Specifically, model 1 tested the effects of total log-in duration; total number of sessions completed (0-8); and covariates, including baseline MVPA, baseline BMI, demographics (income, sex, and age), time-related variables for PA (weekend and season), and a dummy code for the face-to-face group-based treatment (0=control and 1=intervention). Model 2 added engagement with the PA content session (log-in status and duration). Model 3 added engagement with the sedentary behavior session, and model 4 added engagement with the energy balance session. Finally, model 5 included diet-focused content sessions, including the fruits and vegetables, sweetened beverages, and fast food sessions. The log-in status for each content session was a dummy-coded variable (0=no log-in and 1=log-in), and the log-in duration was a continuous variable. In addition, to account for nesting within treatment groups during the face-to-face phase of the program, the mixed modeling approach included a random effect for group-based treatment. Likelihood ratio tests were used to compare the models.

To aid in interpreting the results, all continuous variables were mean centered, including covariates and predictors. Before analysis, outliers for the log-in duration were recoded to reflect a maximum of 3 times the IQR using a winsorizing approach [43]. This resulted in the recoding of <7% of cases. As in previous publications [34,44], missing PA data from the FIT trial were accounted for using a weighted mixed model approach with variance weighting by the inverse of daily wear-time proportions to evaluate the effect of log-in duration on PA [45]. The weighted regression approach is an efficient approach for dealing with missing accelerometry data because cases with a higher proportion of missing wear time are down weighted compared with cases with less proportion of missing wear time, which has been shown to improve the precision in estimating accelerometry-estimated PA [45].

## Results

### Descriptive Statistics

Parents had a mean BMI of 37.50 (SD 9.16) at baseline and an average age of 43.69 (SD 8.94) years and were mostly female (113/119, 95%). On average, adolescents had a BMI percentile of 96.6% at baseline and a mean age of 12.71 (SD 1.67) years and were mostly female (76/119, 63.9%). Table 2 provides participant demographics and baseline MVPA values.

Table 3 provides a summary of the percentage of participants in the web-based tailored intervention who completed each session and the average log-in duration per session. Participants completed an average of 4.25 (SD 3.05) sessions out of the 8 total sessions, including the introductory and closing sessions. On average, participants spent between 728.26 (SD 581.38) and 1246.6 (SD 1025.2) seconds (12.1-20.7 minutes) on each of the sessions. Among the 6 content sessions, sessions on energy balance (56/119, 47.1%), sedentary behavior (42/119, 35.3%), and sweetened beverages (39/119, 32.8%) were completed by the greatest percentage of participants.

**Table 2:** Participant demographics in the tailored web-based intervention (N=119).

Variable				Value
**Adolescent**				
	Sex (female), n (%)		76 (63.9)	
	Age, mean (SD)		12.71 (1.67)	
	Baseline BMI (%)		96.93	
	Baseline MVPA^a^ (square root transformed)^b^		4.15 (1.56)	
**Parent**				
	Sex (female), n (%)		113 (95)	
	Age, mean (SD)		43.69 (8.94)	
	Baseline BMI, mean (SD)		37.50 (9.16)	
	Baseline MVPA (square root transformed)^b^		2.07 (1.24)	
	Married, n (%)		47 (39.5)	
	**Parent education, n (%)**			
		Unreported	3 (2.5)	
		Grades 9 to 11	4 (3.4)	
		Grade 12	14 (11.8)	
		Some college	55 (46.1)	
		4-year college	19 (16)	
		Professional	24 (20.2)	
	**Parent employment status, n (%)**			
		Unreported	3 (2.5)	
		Working	80 (67.2)	
		Unemployed	9 (7.6)	
		Retired	7 (5.9)	
		Disabled	5 (4.2)	
		Homemaker	7 (5.9)	
		Student	3 (2.5)	
		Other	5 (4.2)	
	**Household annual income (US $), n (%)**			
		Unreported	3 (2.5)	
		<10,000	16 (13.4)	
		10,000-24,000	22 (18.5)	
		25,000-39,000	31 (26.1)	
		40,000-54,000	17 (14.3)	
		55,000-69,000	11 (9.2)	
		70,000-84,000	6 (5.0)	
		≥85,000	13 (10.9)	

^a^MVPA: moderate to vigorous physical activity.

^b^MVPA means were weighted by the inverse of daily wear-time proportions.

**Table 3:** Engagement with the tailored web-based intervention sessions (N=119).

Session content	Completed by participants, n (%)	Log-in duration (seconds), mean (SD)
Introduction	96 (80.7)	1246.64 (1025.2)
Energy balance	56 (47.1)	1024.2 (723.67)
Sedentary behavior	42 (35.3)	861.83 (538.22)
Sweetened beverages	39 (32.8)	1021 (608.65)
Physical activity	32 (26.9)	890.26 (552.8)
Fast food	27 (22.7)	944.9 (697.48)
Fruits and vegetables	26 (21.8)	915.06 (619.7)
Closing	65 (54.6)	728.26 (581.38)

### PA Results

#### Parents

In model 1, which included the total log-in duration, total number of sessions completed, and covariates, there was a positive effect of total log-in duration such that parents who spent more time logged in during the 8-week program had greater MVPA (*B*=.001, 95% CI 0.000-0.002; Multimedia Appendix 1). Baseline parent MVPA and income were positively associated with greater MVPA after the web-based intervention, whereas parent age was negatively associated with MVPA. Model 2, which added engagement with the PA session, revealed a negative effect of log-in status for the PA session (*B*=−10.478, 95% CI –16.567 to –4.389) and a positive effect of log-in duration for the PA session (*B*=0.006, 95% CI 0.002 to 0.009). Model 3, which added engagement with the sedentary behavior session, revealed that there was also a positive effect of log-in duration for the sedentary behavior session (*B*=0.006, 95% CI 0.002-0.010), but not log-in status. In model 4, the addition of engagement with the energy balance session did not reveal any additional significant effects. Finally, in model 5, which included the addition of engagement with diet-related sessions, the only significant effect was the log-in status for the fast food session (*B*=6.628, 95% CI 0.967-12.290). Likelihood ratio tests revealed improvements in fit between model 2 and model 1 (*χ*^2^_16_=11.453, *P*=.003) and model 3 and model 2 (*χ*^2^_18_=10.858, *P*=.004), but not between model 4 and model 3 (*χ*^2^_20_=.172, *P*=.92).

In summary, parents who logged into the PA session had lower MVPA, but MVPA increased with greater log-in duration for the PA and sedentary behavior sessions. These associations remained even after accounting for engagement with other content sessions. In addition, logging in to the fast food session was associated with greater MVPA, but the log-in duration for this session was not significantly associated with MVPA. Although model 1 indicated that total log-in duration was positively associated with greater MVPA, this effect was no longer significant when engagement for the different content sessions was included in the model.

#### Adolescents

For adolescents, model 1 revealed a negative effect of total log-in duration (*B*=−0.002, 95% CI −0.003 to 0.000) and a positive effect of total sessions (*B*=2.019, 95% CI 0.176-3.862), such that adolescents displayed greater MVPA after the web-based intervention when their parents completed more content sessions (Multimedia Appendix 2). In addition, baseline adolescent MVPA was positively associated with MVPA after the web-based intervention, whereas weekends were negatively associated, suggesting lower MVPA on weekends (vs weekdays). Unlike parents, adolescents’ engagement with the PA session and sedentary behavior session were not significantly associated with adolescent MVPA, as revealed by models 2 and 3. In addition, models 4 and 5 revealed that engagement with the sessions on energy balance, fruits and vegetables, sweetened beverages, and fast food was not significantly associated with adolescent MVPA after the web-based intervention. Likelihood ratio tests revealed that the fit did not significantly improve across any of the models. In summary, engagement with the different content sessions was not significantly related to MVPA after the web-based intervention among adolescents. Although model 1 indicated a negative effect of total log-in duration and a positive effect of total sessions completed, these effects were no longer significant after accounting for engagement with the different content sessions.

## Discussion

### Principal Findings

This study investigated whether engagement with different tailored behavioral sessions, measured in terms of log-in status and duration, was associated with improvements in MVPA among African American families. Among parents, logging into the PA session was not associated with greater MVPA. However, consistent with our hypothesis, MVPA did increase with greater log-in duration for the PA and sedentary activity sessions, suggesting that greater exposure to the tailored PA-related content had a positive impact on parents’ MVPA. Surprisingly, the results also indicated that logging into the fast food session was associated with greater MVPA, but log-in duration for the fast food session was not significantly associated with parent MVPA. Consistent with past reviews, which have found that the clustering effects of PA and diet are complex [46,47], this finding may suggest that an interest in learning more about reducing fast food consumption correlates with PA engagement but that greater exposure to tailored fast food–related content does not promote greater PA.

The results also indicated that engagement with different behavioral content sessions was not significantly related to MVPA after the web-based intervention among adolescents, suggesting that the effects observed in parents did not carry over to their adolescent children. Although parent involvement is viewed as an important component for adolescent eHealth interventions [48,49], past eHealth interventions using parents as the agent of change have yielded weak or nonsignificant effects on adolescents’ PA and BMI [22]. These findings suggest that it may be important to directly target both parents and adolescent and improve the quality and duration of eHealth interventions to improve their efficacy [22]. Although engaging youth in eHealth interventions remains a major challenge, future interventions might consider improving engagement by co-designing digital interventions with input from adolescents and enhancing personalization through additional tailoring [50]. In a follow-up study, we conducted individual interviews with adolescents from the FIT trial and found that they experienced numerous sources of stress, which in some cases, promoted unhealthy behaviors (eg, sedentary behavior) and avoidant coping [51]. Thus, to improve engagement among adolescents in eHealth interventions, future researchers should consider integrating tailoring around barriers related to stress and coping directly for adolescents (not just parents) to enhance families’ capacity to engage in sustainable health behavior change.

### Implications for Future Research

This study has several important implications for future tailored web-based interventions. First, this is one of the first studies to demonstrate a behavior-specific pathway between engagement with tailored content and behavioral outcomes. Although previous studies have highlighted the importance of adherence by evaluating engagement at the aggregate level (eg, total log-in frequency or dose) [31,32], few studies have evaluated engagement with different types of tailored content and associations with health behavior change. In this study, we were able to distinguish between engagement with different types of content (eg, PA vs diet), but not between components within each session, such as engagement with the behavioral content versus positive parenting practices. Thus, future web-based studies may benefit from integrating program use data that captures engagement with different types of tailored components. One approach to better isolate the role of engagement with different types of tailored content is through a microrandomized trial in which participants are randomized across time to receive different types of tailored messages [52], rather than manipulating several tailoring variables simultaneously as in this trial.

Second, we found that although parents were more likely to complete some sessions (eg, energy balance, sedentary behavior, and sweetened beverages), a lower percentage of participants completed the fast food and fruit and vegetable sessions. Future research is needed to further understand the barriers related to engagement with these weight-related behaviors among African American families, especially regarding structural barriers related to cost and access to healthy foods [53]. Although previous interventions have primarily focused on individual- and family-related barriers to healthy eating, future studies may benefit from using tailoring to address broader neighborhood factors. For example, for some African American families, it may be important to tailor information on healthy eating to address barriers related to convenience and time, whereas for others, tailoring around cost and access may be more relevant [54,55].

### Strengths and Limitations

This study has several strengths, including the use of a multitheoretical framework, deep-level cultural tailoring, an underrepresented racial minority sample, and an objective measure of PA. The study design allowed us to compare the effects of engagement with different types of tailored behavioral sessions (eg, diet- vs PA-related contents). However, 1 limitation is that our study design did not allow us to differentiate the unique or additive effects of different types of tailoring (eg, tailoring around parentings vs deep-level cultural factors). Furthermore, because the web-based comparison program included links to external websites, we were unable to measure the log-in duration in a comparable manner with the web-based intervention. Decisions around selecting an appropriate comparator remain a central issue in the literature on tailored interventions [56-58]. To better understand the effects of different tailoring constructs, future research may consider using microrandomized designs that allow for the systematic testing of different tailored content and features. Furthermore, rather than comparing tailored interventions with a nontailored health education program, a more robust test for future research to consider is a matched/mismatched design in which participants are randomized to receive either a message that is tailored to them or an alternative message that they would not be assigned to receive if the message was chosen based on their data. This design allows us to test whether tailoring improves outcomes, whether each message outperforms other messages on average, and whether some messages produce stronger effects when tailored messages are used than when other messages are used.

Another limitation of this study is that adolescents were not directly involved in the web-based intervention, which may have weakened the overall impact of the intervention. Despite these limitations, the web-based tailored intervention integrated several novel tailoring constructs, including expanding upon the use of positive parenting practices and deep-level cultural tailoring, and found positive effects of engagement on parent MVPA. Although previous tailored studies have drawn from a relatively narrow range of theories and constructs [56-58], this study provides an example of how to integrate additional motivational and behavioral theories in the context of positive parenting practices.

### Conclusions

The results of this study highlight the need to disentangle the impact of engagement with different tailored content to improve the efficacy of web-based tailored interventions, especially for improving PA in African American families. Among parents, MVPA did increase with greater log-in duration for the PA and sedentary activity sessions, suggesting that greater exposure to the tailored PA-related content had a positive impact on parents’ PA. Future web-based studies may benefit from integrating program use data that captures engagement with different types of tailored theoretical construct components.

## Appendix

Multimedia Appendix 1 Fixed effects of log-in status and duration on moderate to vigorous physical activity for parents (N=119).

Multimedia Appendix 2 Fixed effects of log-in status and duration on moderate to vigorous physical activity for adolescents (N=119).

## Abbreviations

FIT: Families Improving Together
M+FWL: Motivational and Family-Based Weight Loss intervention
MVPA: moderate to vigorous physical activity
PA: physical activity
