# Supplementary figures and images for: Engagement With Tailored Physical Activity Content: Secondary Findings From the Families Improving Together for Weight Loss Randomized Controlled Trial
Source: J Med Internet Res. 2023 Apr 12;25:e42581. doi: 10.2196/42581 (PMC10134014; doi:10.2196/42581)

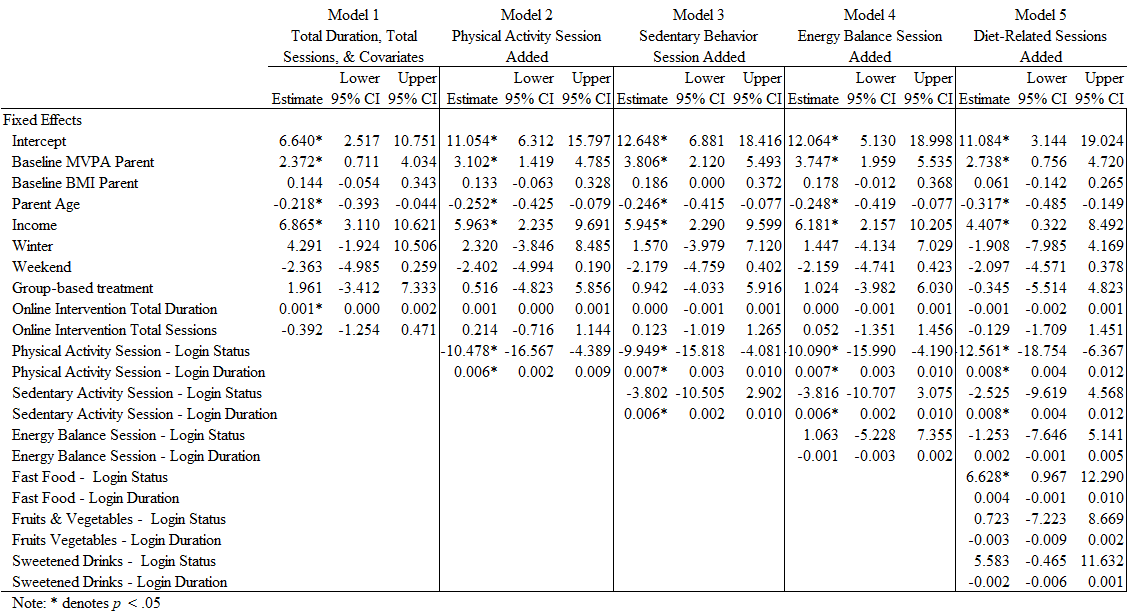

Supplement: Multimedia Appendix 1 [file jmir_v25i1e42581_app1.png]

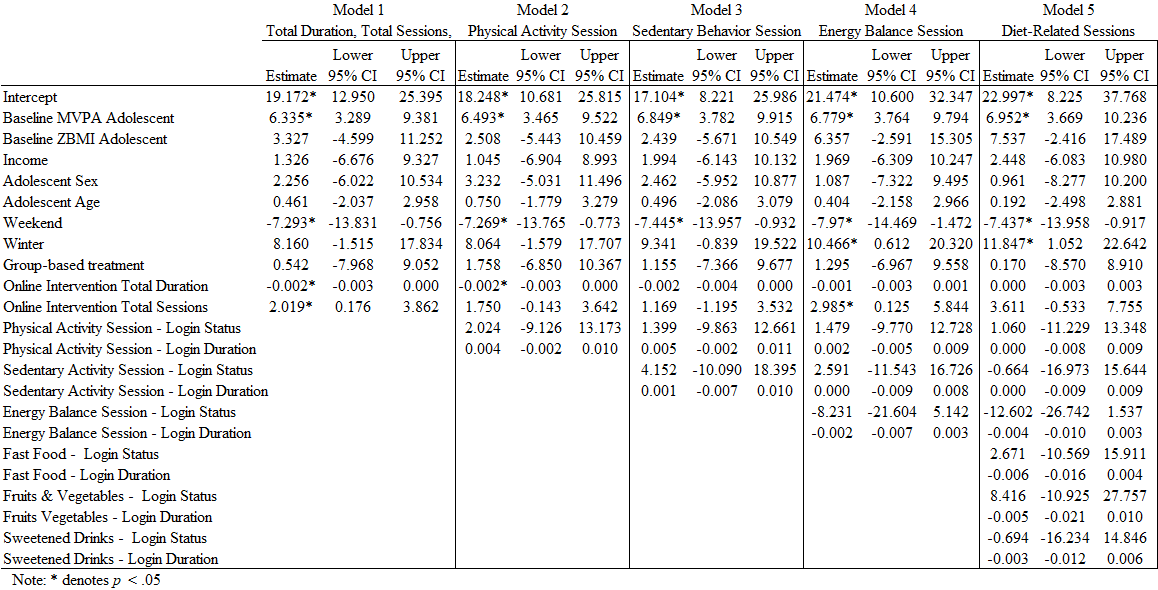

Supplement: Multimedia Appendix 2 [file jmir_v25i1e42581_app2.png]
